# Increased mortality with the use of adrenaline in shock: the evidence is still limited

**DOI:** 10.1186/s13054-016-1465-4

**Published:** 2016-09-20

**Authors:** Rodrigo Antonini Ribeiro, Luciane Maria Fabian Restelatto

**Affiliations:** 1 IMED – Faculdade Meridional, School of Medicine, R. Sen. Pinheiro, 304 - Vila Rodrigues, Passo Fundo, RS 99070-220 Brazil; 2 Graduate Program in Epidemiology, Universidade Federal do Rio Grande do Sul, Porto Alegre, RS Brazil; 3 Hospital de Clínicas de Porto Alegre, Porto Alegre, RS Brazil

We have read with great interest, but also with some concern, the paper by Tarvasmäki et al., recently published in *Critical Care* [1]. In this observational study, adrenaline was independently associated with mortality in cardiogenic shock (CS). The data presented are the odds ratio (OR) of a multivariable propensity score-adjusted analysis, where patients that used adrenaline had an adjusted OR of 3.0 (95 % confidence interval 1.3–7.2) for 90-day mortality in comparison with patients who had not received adrenaline.

The main concern with the article is precisely the choice of the OR as its measure of effect. The misleading use of OR as an approximation of the relative risk (RR) when the outcome of interest is very frequent (which is certainly the case in this study, where the mortality incidence was around 40 %) is a long recognized problem and alternative methods for analysis, such as the Poisson regression with robust variance, have been suggested as appropriate approaches to estimate the RR for more than a decade [2]. Since the authors did not present the RR, one could use a suggested well-known formula [3] to estimate the RR from a given OR and outcome incidence: RR = OR/(1 − Incidence + (Incidence × OR)). Applying this equation, the RR for 90-day mortality would be 1.67 (1.16–2.07).

Although this number still shows a statistically significant increase in mortality, one must remember that this information comes from an observational study. The GRADE working group proposal for rating the quality of evidence for interventions ranks the conclusions from observational studies as low quality evidence [4]. This quality of evidence can be rated up if a large magnitude of effect is present. However, the suggestion in the GRADE guidelines is that this rating up should be done when the RR as at least equal to 2, which was not the case in this study [5]. In conclusion, the evidence suggesting an increased mortality risk associated with adrenaline use in CS is low quality (at best, since one could rate it down to very low quality if we consider that there is imprecision in the estimate) and, therefore, an adequately designed and powered clinical trial is imperative to provide a reliable answer to this question.

## Abbreviations

GRADE: Grading of Recommendations Assessment, Development and Evaluation
OR: Odds ratio
RR: Relative risk
