# Supplementary material for: Increased mortality with the use of adrenaline in shock: the evidence is still limited
Source: Crit Care. 2016 Sep 20;20:289. doi: 10.1186/s13054-016-1465-4 (PMC5028931; doi:10.1186/s13054-016-1465-4)
Supplement: Related texts

# Reply: Authors’ response to “Increased mortality with the use of adrenaline in shock: the evidence is still limited”

**Authors:** Tuukka Tarvasmäki, Reijo Sund, Johan Lassus, Alexandre Mebazaa, Veli-Pekka Harjola

We thank Ribeiro and Restelatto for their comments on our article about assessing the concurrent use of vasoactive medications in cardiogenic shock [1]. They raise two potential issues regarding our main finding, the association between adrenaline use and 90-day mortality: the use of the odds ratio (OR) instead of relative risk (RR) and that the evidence is from an observational study. While we agree that the OR might not always accurately describe the RR, logistic regression analysis is a gold standard statistical technique for binary responses, so it was an obvious choice also in our study. Moreover, after conversion from ORs, both the univariate and adjusted multivariable RR for adrenaline use were over 2 (2.3 and 2.2, respectively).

As we were curious about the association between adrenaline use and increased mortality, we performed and reported further analyses using additional propensity score adjustments in the subgroup of vasopressor-treated patients as described in our article. The effect size diminished a bit but remained statistically significant (OR 3.0, 95 % confidence interval 1.3–7.2, *p* = 0.01). We also reported analyses using propensity score matching, Kaplan–Meier curves and log-rank tests, as well as an adjusted Cox regression model and the association was statistically significant regardless of the analysis technique. As suggested by Ribeiro and Restelatto, we performed an additional analysis using Poisson regression with robust variance: for adrenaline use in a multivariable model with propensity score adjustment within the subgroup of vasopressor-treated patients, the RR for 90-day mortality was 1.5 (95 % confidence interval 1.1–2.1, *p* = 0.017), i.e., still a statistically significant association.

So, the main finding is supported by several sensitivity analyses and we can be confident that there is a clearly statistically significant association between adrenaline use and 90-day mortality in our data. Albeit being a result from an observational study, we argue that this is still a clinically significant finding, especially considering the very high frequency of (poor) outcome also emphasized by Ribeiro and Restelatto. This warrants attention because it raises justified questions about the safety of this treatment. And we fully agree, as we conclude in our article, that there is a need for randomized controlled trial to confirm this detected association.
